# A study on the influence of service robots’ level of anthropomorphism on the willingness of users to follow their recommendations

**DOI:** 10.1038/s41598-022-19501-0

**Published:** 2022-09-10

**Authors:** Elahe Abdi, Dewi Tojib, Alexander Kenwa Seong, Yamika Pamarthi, George Millington-Palmer

**Affiliations:** 1 Department of Mechanical and Aerospace Engineering, Monash University, Melbourne, 3800 Australia; 2 Department of Marketing, Monash University, Melbourne, 3800 Australia

**Keywords:** Electrical and electronic engineering, Mechanical engineering, Human behaviour

## Abstract

Service robots are increasingly deployed in various industries including tourism. In spite of extensive research on the user’s experience in interaction with these robots, there are yet unanswered questions about the factors that influence user’s compliance. Through three online studies, we investigate the effect of the robot anthropomorphism and language style on customers’ willingness to follow its recommendations. The mediating role of the perceived mind and persuasiveness in this relationship is also investigated. Study 1 (*n* = 89) shows that a service robot with a higher level of anthropomorphic features positively influences the willingness of users to follow its recommendations while language style does not affect compliance. Study 2a (*n* = 168) further confirms this finding when we presented participants with a tablet vs. a service robot with an anthropomorphic appearance while communication style does not affect compliance. Finally, Study 2b (*n* = 122) supports the indirect effect of anthropomorphism level on the willingness to follow recommendations through perceived mind followed by persuasiveness. The findings provide valuable insight to enhance human–robot interaction in service settings.

## Introduction

The service sector is being revolutionised by the integration of service robots. The deployment of such robots in service establishment has been explored in numerous industries including hospitality, education, healthcare and aged care; aiming to identify the best combination of physical and social characteristics that produce the greatest affinities towards a mechanical entity^1–3^. These social robots with aspects of human intelligence have indeed transformed human–human interactions into self-service interactions in many parts of the industry^4^. With the ability to adapt and carry out a “complex series of actions”, these robots are slowly replacing human employees in the service sector^5^. To further assimilate service robots within both commercial and domestic spheres, researchers explore the advantages of human–robot interactions (HRI)^6^. Reducing the fatigue of human labour, increasing the precision of the task and minimising repetitive tasks^7^ whilst ensuring the regularity of work without strikes^6^ or avoiding disruption in pandemics are some of the positive aspects of employing service robots.

To further improve the relationship between consumers and service robots, research has focused on the customer experience in interaction with these robots^5^. This interaction may be improved by modulating the anthropomorphic qualities of a robot and its language style. Previous research explored the influence of robot appearance, voice and behaviour on customers, and has already paved the way to increasing the positive interactions between customers and service robots^8^. However, the effect of these factors on the customer’s willingness to follow the robot’s recommendations and the process that underlies this proposed relationship are yet to be explored. This knowledge will add a significant value to our understanding of human–robot interactions.

Therefore, this study aims to investigate the influence of the different levels of anthropomorphism of the service robots and their language style on the participant’s willingness to follow their recommendations. User’s perception of the service robot (i.e. perceived mind) and its persuasiveness are further examined as the underlying mechanism in the relation between the levels of anthropomorphism of the robot and participants’ willingness to follow its recommendations. To achieve the goals of this research, we conducted three online studies in which participants interacted with a service robot in virtual reality context. We report the results, discuss the findings and highlight the limitations of this study, and conclude with the implications of our discoveries for the service sector.

## Theoretical background and hypotheses

### Anthropomorphism

Humans often tend to anthropomorphise objects in everyday life^9,10^. Whether customers see the product as humanlike primarily depends on the presence or absence of certain “features that convey a sense of humanity” such as emotional face gestures. For example, a smiling car would be evaluated more positively compared to a frowning one^11^. Hence, during an interaction with an object, including a service robot, customers intuitively attribute humanlike qualities to it^12,13^. As social robots, service robots are often distinguished as one of the following three types based on their anthropomorphic features: (1) mechanoids that are predominantly machine-like and devoid of human characteristics; (2) humanoids that are not overtly human but embody human-like attributes including bodies and facial features; (3) androids which are machines which mirror humans as closely as technically possible^14^. User’s motivation to understand the behaviour of these service robots and their desire for social contact are among other factors affecting anthropomorphism^15^. This highlights that anthropomorphising service robots is a result of human perception which can influence customers’ behaviours.

Investigating the anthropomorphic features implemented in service robots is quite common in HRI. Research indicates that the success of social robots cannot solely be measured by the quality of their electromechanical components or by their performance criteria^16^. Unlike early industrial robots whose success was measured based on their compliance with quality standards, social robots’ success is measured by the satisfaction of the consumers. To determine and improve the satisfaction levels in customers, it is necessary to investigate human perception of social robots. This perception is widely influenced by the physical design of the robot or the degree to which it looks and acts like a human^17,18^. Physically, this includes developing robots that exhibit human-like characteristics such as face and voice^19^ to provoke feelings of familiarity and similar positive emotional responses^20^. These pre-existing expectations on the appearance or physical design can be applied to service robots and affect human experience.

The anthropomorphic attribute of voice is directly related to the robot’s communication style. Previous research has investigated the effect of social feedback^21^, politeness^22^, and storytelling^23^ on the perceived persuasiveness of the robot and customer’s satisfaction. A consumer’s behaviour is susceptible to change when spoken to in their native language compared to their second language^24^. This indicates that the robot’s anthropomorphic attribute of vocal communication can be influential in customer’s willingness to follow its recommendations. Additionally, associating a literal (vs. figurative) style of speech with a higher level of social agency is also symbolic of higher knowledgeability of the robot and is more effective in satisfactory customer evaluation^25^. Similarly, another study discovered that literal language had more persuasive power than figurative language, as it highlighted the reviewer’s high expertise^26^. Literal language was defined as using words with their exact meanings, while figurative language was defined as using metaphors and wordplay to achieve new or more complicated understanding. In contrast, a study on service referrals found abstract (vs. concrete) language to be more persuasive, signifying high prior knowledge of the recipient^27^. Abstract language was defined as using adjectives to generalise a behaviour (e.g., to be aggressive), and concrete language was defined as descriptive action verbs referring to observable behaviours (e.g., to hit somebody). Therefore, the persuasiveness of the language style is dependent upon human perception and the overall service context. In this paper, the terms informative and emotive language are used to describe literal and figurative language styles respectively.

It has been shown that a combination of the mentioned attributes is required to reinforce the perception of anthropomorphism^28^. Although the effect of the *level of anthropomorphism* on the general perception of the customer of the service robot has been investigated in multiple studies, its effect on their willingness to follow the robot’s recommendations has not been examined. In addition, while the influence of *communication style* as a subset of anthropomorphism on persuasiveness has been investigated in literature, the combined effect of *language style* and *robot type* on customers’ willingness to follow recommendations is unclear.

### The role of perceived mind and persuasiveness

Service robots have long been perceived only as tools that carry out certain tasks and do not generally receive the same respect as human beings^29^. However, with increased use of social robots in recent years, humans began to perceive robots as having minds^30^. This is known as mind perception and typically involves attributing mental abilities to non-human entities^31,32^. Previous research conducted on mind perception discovered that this concept has two dimensions: agency (“to do”) and experience (“to feel”)^33^. Some of the capacities that defined experience were joy and desire. While the agency was depicted by morality, memory, emotion recognition, communication and thought. The performance of the robot, social factors and conventions are among the factors that affect user’s trust on a robot^34^. The ability of the robot to understand the beliefs and intentions of others, known as the theory of mind, positively affect this^35^. User’s trust towards the robot can be further affected by its physical appearance and reliability^36^.

Service robots are attributed to having a higher level of cognition (agency) compared to experience^13,37^. The theory that plays a significant role in this skew between the two dimensions of mind perception is, once again, the theory of anthropomorphism of robots. When robots display more human-like attributes, they are perceived as having higher mental capacities. Simple movements that symbolise humanness include blinking, eye contact, gestures or changing facial expressions. Such actions implemented in a robot attract perceptions of increased intelligence and hence a higher level of mind, during human–robot interactions^38–40^. Other research, discovered that actions like hand-waving and self-introducing performed by a robot, can increase the perception of mind^32^. This is because such behaviour is often human specific and not common in machines. Hence, the robot’s capacity to mimic human-like actions indicated a high perception of mind in the participants.

A product of increased perception of mind can be persuasion, which plays a major role in influencing the user’s decisions. It has been identified that higher levels of social capability are depicted through nonverbal gestures and gaze of the robot, persuading the users to follow its recommendations^41^. Also, combined demonstration of attributes such as gaze and gestures makes the robot more persuasive than displaying any of these attributes alone^42^.

Hence, the anthropomorphic features can considerably alter the robot’s persuasiveness^43^. Customers may perceive different qualities, such as knowledgeability and sociability in robots based on the appearance^44^ of the robot and this may influence the customer’s willingness to interact with the robot again^45^. The name and voice of the robot as well as the task that it undertakes affect the user’s perception^46^. However, the influence of robot’s perceived gender on HRI varies in different studies^47,48^ depending on the user’s gender-related characteristics and pre-experimental attitudes among others^49^. Users’ perception, including their trusting beliefs and liking towards the robot positively affect the acceptance of the robot and its persuasiveness^50^.

Therefore, user’s *perception* of service robots is a key deciding factor in their willingness to continue interacting with the robot in the future. This perception seems to be influenced by language style and the anthropomorphic features of the service robot.

### Study design and hypotheses

In summary, previous research has identified the effect of robot type and language style in improving the overall customer experience in HRI, but the *factors* and *mechanisms* affecting their willingness to follow the robot’s recommendations are yet to be fully explored.

Robot type and language style are widely accepted as two of the factors that affect customers’ experience in HRI. The first step in manipulating these variables in HRI design is to ensure they represent what they are intended to. Thus, the research questions we address in this paper are *‘Which type of service robot (low anthropomorphised vs. high anthropomorphised) and language style (informative vs. emotive style) play a more important role in compliance?’* and *‘What is the underlying mechanism that explains the relationship between service robot types (low anthropomorphised vs. high anthropomorphised) and language style (informative vs. emotive style) with compliance?’.*

Based on the literature, there is evidence supporting the positive effect of some anthropomorphic features on the users’ experience. However, there has been inconsistencies in findings regarding the effect of language style^26,27^. Thus, we expect robot type to influence the users’ willingness to follow recommendations but do not have any predictions about the effect of language style. Note that in this paper we aim to investigate the influence of the level of service robot anthropomorphism—through a combination of attributes as summarised in 2.1—on users’ willingness to follow recommendations rather than the individual attributes of such robots, that affect the anthropomorphism itself. Hence, we hypothesise:

#### H1

A service robot with a higher level of anthropomorphism positively influences the willingness of users to follow its recommendations.

To understand the mechanism behind H1 and based on the literature, we hypothesise:

#### H2

The positive effect of the service robot’s level of anthropomorphism on the user’s willingness to follow its recommendations is mediated by perceived mind and persuasiveness.

Three scenario-based online studies are conducted to answer the research question and test our hypotheses. Study 1 aims to address the first research question and tests H1. Study 2a validates the effect of service robot’s level of anthropomorphism on users’ willingness to follow its recommendation controlling for the embodiment of service robot and its communication style. Study 2b aims to answer the second research question and tests H2.

## Method and results

### Experiment setup

A virtual environment was created using the Unity 3D engine to model a hotel lobby. Participants interact with this simulated environment on the Zoom video conferencing platform in Study 1 and Study 2b, and through recorded videos of the interaction in Study 2a. Previous research has shown that in many cases having the actual physical robot is not necessary for HRI studies, highlighting the advantages of simulated interaction in recruiting more participants as well as their flexibility in replicating a range of scenarios^51^. This approach is specifically useful in this research where the HRI happens in a hotel lobby and access to such physical environment is specifically limited during the COVID-19 pandemic.

All studies reported in this paper were approved by the Human Ethics Low Risk Review Committee at Monash University (ID: 21939). All methods were performed in accordance with the relevant guidelines and regulations. Only adult participants capable of consenting for themselves were recruited. In Studies 1 and 2b, all participants read an explanatory statement describing what the study involves. They then signed a consent form to show that they agreed to all terms and conditions. In Study 2a, having read the explanatory statement, all participants provided implied consent when completing the online study. Their participation was voluntary.

### Robots modelled in the experiment

**Figure 1:**
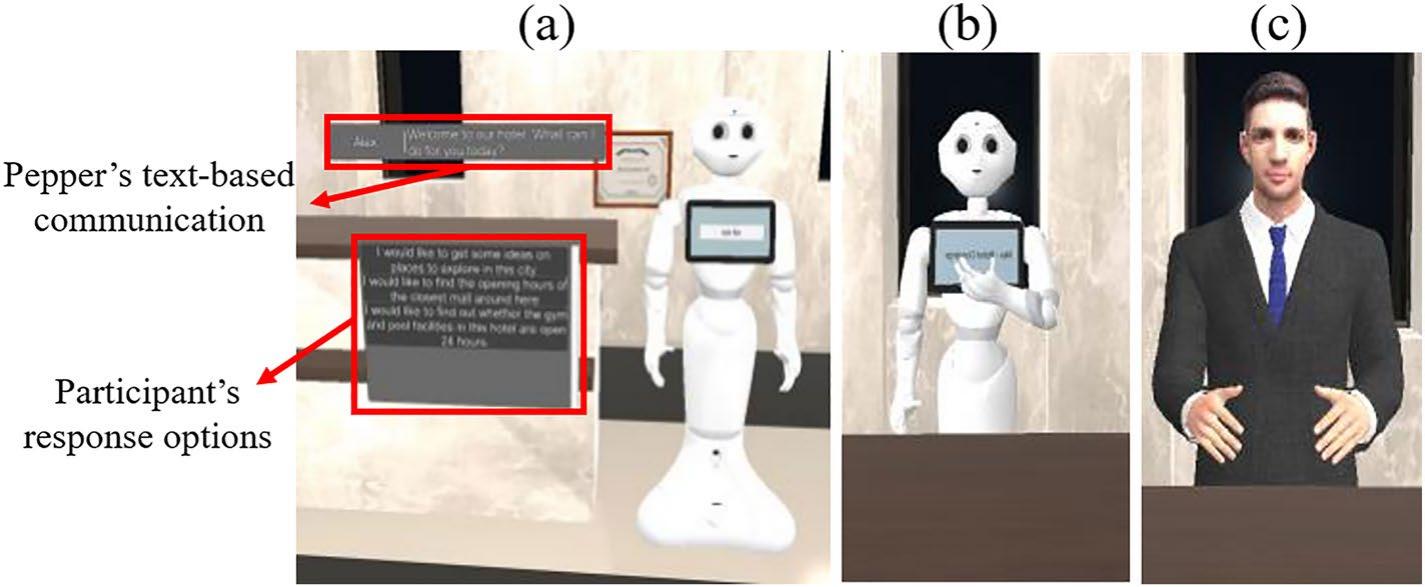
Service robots with three different levels of anthropomorphism. (**a**) Pepper robot communicating with text, (**b**) Pepper robot communicating with voice and gestures, (**c**) android communicating with human-like voice, gestures and appearance.

Participants engaged with one of the following three types of robot as the concierge: (i) the Pepper robot, by SoftBank Robotics^52^ interacting with the customer solely through its tablet (PT), (ii) the Pepper robot as a humanoid using its voice, hand gestures and head movements (PV), and (iii) the android with a human-like voice and appearance but slightly robotic movements (AN) (Fig. 1).

The three PT, PV and AN robots exhibit different levels of anthropomorphic behaviour and appearance (Table 1). While the Pepper robot is a humanoid, it is visually less anthropomorphic compared to the android. Both the Pepper robot and the android are largely perceived as male based on previous research^53^ and their visual characteristics, respectively. PT exhibited the lowest level of agency as it did not have any movements or gestures and only communicated via the written text on the screen. PV and AN both used similar head and hand movements during the conversation^40^. AN also used facial gestures like smile and frown. PV communicated with a robotic voice, while AN used a human-like voice^54^. A reference study^55^ utilised a similar approach of manipulating low and high anthropomorphism robots.

**Table 1:** The manipulated attributes of PT, PV, and AN to represent different levels of anthropomorphism.

Manipulated attribute	PT	PV	AN
Physical embodiment	Humanoid	Humanoid	Android
Communication style	Text	Humanoid Voice	Android Voice
Movement	No	Head, hand movement	Head, hand movement
Facial gestures	No	No	Yes (limited)

### Study 1

#### Method and procedure

This study utilized a 3 (robot type: PT vs. PV vs. AN) × 2 (language style: emotive vs. informative) between-subjects experimental design.

We recruited 89 participants over 18 years of age through flyers distributed within Monash University, Australia and invitations on social media (*M*_*age*_ = 28.9 years, *SD* = 10.19, 56.2% male). Participants first completed a pre-quiz where they answered demographic-related questions (e.g., age, gender). They were then asked to read and imagine a scenario whereby they played a role as a tourist visiting a new city and spoke with a hotel concierge to identify the best sightseeing attractions for a one-day trip.

**Figure 2:**
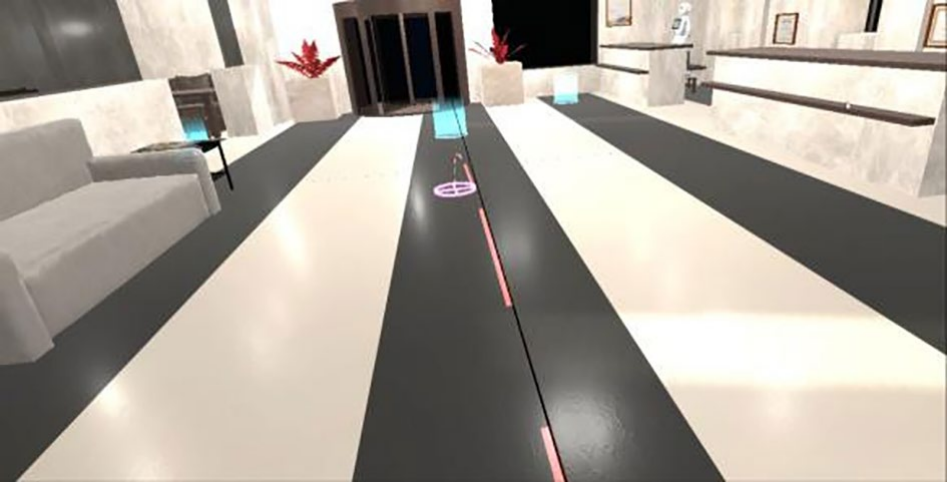
Virtual hotel lobby where the hotel concierge interacts with the customer.

Afterwards, the supervisor would run the virtual environment simulating the hotel lobby from the executable program compiled from the Unity project (Fig. 2). The supervisor would then share their screen and enable remote control on Zoom for the participant to interact with the service robot that plays a role as the hotel concierge. The supervisor randomly allocated participants into one of the three service robots under investigation. The supervisor would place the participant in front of the service robot called Alex. This would trigger the conversation with the robot. The participant was then able to pick their responses within the conversational interaction by using their own mouse to select their responses. During their interaction, the supervisor randomly allocated participants into one of the two language styles used by the service robot when providing information on three fictional touristic locations upon request. Upon finishing the interaction, the supervisor would end their screen sharing, and the participant would complete the post-quiz where they answered the remaining items related to dependent and mediating variables. Appendix A describes the interaction scenario in detail.

#### Measures

Participants were asked to indicate their agreement to one item measuring their likelihood to follow recommendation by the service robot (*M* = 5.51, *SD* = 1.31). To validate our service robots’ anthropomorphic manipulation, participants indicated whether the robot they interacted with felt like a person using one item adopted from (Kim, Schmitt et al. 2019) (*M* = 3.89, *SD* = 1.73). Then, they evaluated the warmth of the robot as another indicator of anthropomorphism using five items adapted from^56^ (*M* = 4.86, *SD* = 1.22, *α* = 0.89). To validate our service robots’ language style manipulation, participants were asked to indicate the extent to which the service robot communicated with more informational or emotional language style (1 = strongly informational to 7 = strongly emotional) using one item adapted from^57^ (*M* = 3.42, *SD* = 1.98). All the other items were measured on a seven-point scale (1 = Strongly Disagree to 7 = Strongly Agree). Participants were also asked to rate whether they believed that the scenario presented to them was realistic (1 = Not at all realistic, 7 = Very realistic*; M* = 5.56, *SD* = 1.18), with no difference between conditions (*F* (2, 83) = 2.63, *p* = 0.08). Appendix B lists all items used in this study. Note that the aim of Study 1 was to check H1 and the perceived mind and persuasiveness measures presented in Appendix B were only used in Study 2a and Study 2b. For constructs that were measured by multiple items, we created an overall index by taking the average of all items measuring that particular construct and used this overall index in our data analysis.

#### Analysis and results

##### Manipulation check

A one-way ANOVA revealed a significant difference between the two language *style* conditions, with participants in the informative language style condition reporting a lower score in perceived language style than those in the emotional language style condition (*M*_*informational*_ = 2.23, *M*_*emotional*_ = 4.58, *F*(1, 86) = 48.75, *p* < 0.001); confirming the successful manipulation of language style of the service robot.

A one-way ANOVA revealed that participants reported significantly different levels of perceived anthropomorphism (robot felt like a person) for the three service robot behaviour stimuli (F (2,85) = 12.57, p < 0.001). Pairwise comparisons indicated that the mean score for AN (*M* = 5.03, *SD* = 1.45) was significantly different from PT (*M* = 3.41, *SD* = 1.72) and PV (*M* = 3.22, *SD* = 1.45). However, PT was not significantly different from PV. In addition, a one-way ANOVA further revealed that participants reported significantly different levels of perceived warmth as an indicator of anthropomorphism for the three service robot behaviour stimuli (F (2,85) = 11.76, p < 0.001). Pairwise comparisons indicated that the mean score for AN (*M* = 5.65, *SD* = 0.94) was significantly different from PT (*M* = 4.52, *SD* = 1.25) and PV (*M* = 4.41, *SD* = 1.08). However, PT was not significantly different from PV. Taken together, these results suggest that participants perceived the service robots in PT and PV evoke similar level of anthropomorphism. Considering PV, the Pepper robot moves and talks with participants during an interaction but still was perceived as similar in the anthropomorphism level as PT, we believe PV is better in representing a low anthropomorphised service robot and more comparable to AN. Hence, in the further analysis, we only considered two conditions represented by PV to represent a low anthropomorphised service robot and AN to represent a high anthropomorphised service robot.

##### Hypothesis testing

A two-way ANOVA revealed a non-significant main effect of language style (*F*(1, 58) = 0.44, *p* = 0.51) and a significant main effect of service robot type (*F*(1, 58) = 5.69, *p* = 0.02). A non-significant interaction effect of language style and service robot type on likelihood to follow recommendation was also detected (*F*(1, 58) = 0.75, *p* = 0.39). Participants who were served by the android robot (high anthropomorphised robot) exhibited a higher likelihood to follow the service robot’s recommendation compared to those who were served by the Pepper robot (low anthropomorphised robot) (*M*_*PV*_ = 5.19, *M*_*AN*_ = 5.97, *p* = 0 0.02), providing support for H1.

### Study 2a

In Study 1, we presented participants with service robots that vary in their appearance and behaviours (PT, PV, and AN) following an approach of anthropomorphism manipulation by^55^ as well as their language and communication style. Considering we found no differences between PT and PV, and to control for the possible confounding effect of language/communication style and embodiment condition, it is of interest to further investigate whether our findings remain valid where the service robot does not have an anthropomorphic appearance and communicates with the exact same language and communication style as the AN. In our study, we followed^58^ who presented no anthropomorphic embodiment in the form of a tablet screen. In addition, since we found no effect of language style, we chose only the informative language style for Study 2a, and instead tested communication style (text vs. voice). Hence, we conducted an additional study that manipulates service robot embodiment (tablet screen vs. android) and communication style (text vs. voice). Consistent finding with Study 1 provides greater confidence in proposing the positive effect of robot anthropomorphism on users’ compliance.

#### Method and procedure

We recruited 198 US participants from Amazon Mechanical Turk (MTurk), a US-based consumer research panel. We sent an invitation to participate in this study only to those who are at least 18 years old. 30 participants failed an attention check resulting in 168 usable results for our main analysis (*M*_*age*_ = 40.3 years, *SD* = 11.17, 48.2% male). This online study utilized a 2 (embodiment: tablet screen vs. android robot) × 2 (communication style: text only vs. voice only) between-subjects experimental design. The language style used in both conditions was informative. In the text condition, the text appears in a speech bubble next to the concierge. In the voice condition, the tablet and the AN used the same human-like voice as in Study 1.

Similar to Study 1, participants were first asked to complete demographic-related questions (e.g., age, gender). They were then asked to read and imagine the same scenario as before. They went through the same step-by-step processes as participants in the previous study; however, as this study is an online study, participants were asked to watch a series of videos showing the behaviours of the hotel concierge. Video-based studies have been shown to be effective in eliciting comparable behaviours^59^ and perceptions^60^ in remote participants, while allowing data collection from a larger number of participants within a fraction of time. These videos were produced from the virtual environment specifically developed for the previous two studies; hence, the conversation and the movements of the AN shown in these videos were exactly the same as in Study 1. Note that the voice communication style for the tablet included in Study 2a did not appear in Study 1. Participants interacted with the hotel concierge through answering questions embedded in the questionnaire. After viewing these videos, they were then asked to complete the remaining questions. Each participant was randomly allocated to one of the four possible conditions.

#### Measures

In this study, we manipulated embodiment of service robots, i.e., the appearance of service robots in the eyes of users. Similar to previous studies, to validate our embodiment manipulation, participants answered the same anthropomorphism items that we used in previous studies. First, they indicated whether the concierge they interacted with felt like a person (*M* = 4.30, *SD* = 1.76). Participants were then asked to evaluate the warmth of the concierge as another indicator of anthropomorphism (*M* = 5.16, *SD* = 1.28, *α* = 0.91). Participants were asked to indicate their agreement to one item measuring their likelihood to follow recommendation by the concierge (*M* = 5.72, *SD* = 1.15). Furthermore, as we see this study as the bridging study to test H2, participants were also asked to indicate their perceived mind towards the concierge using four items adapted from^33^ (*M* = 3.97, *SD* = 1.61) and their evaluation towards the concierge’s persuasiveness using one item (*M* = 5.15, *SD* = 1.36). Participants were also asked to rate whether they believed that the scenario presented to them was realistic (*M* = 5.56, *SD* = 1.10), with no difference between conditions (*F*(1, 164) = 0.12, *p* = 0.73). Appendix B lists all items used in this study.

#### Analysis and results

##### Manipulation check

A one-way ANOVA revealed that participants reported significantly different levels of perceived anthropomorphism (concierge felt like a person) for the AN and tablet (F (1,167) = 182.63, p < 0.001). Pairwise comparisons indicated that the mean score for AN (*M* = 5.57, *SD* = 0.87) was significantly different from tablet screen (*M* = 3.04, *SD* = 1.48). A one-way ANOVA further revealed that participants reported significantly different levels of perceived warmth as an indicator of anthropomorphism for the AN and tablet. Participants interacting with the tablet screen reported a lower score in perceived anthropomorphism than those interacting with the AN (*M*_*T*_ = 4.67, *M*_*AN*_ = 5.65, *F*(1, 167) = 28.33, *p* < 0.001); confirming the successful manipulation of embodiment.

##### Hypothesis testing

A two-way ANOVA revealed a non-significant main effect of communication style (*F*(1, 164) = 0.08, *p* = 0.78) and a significant main effect of service robot type on likelihood to follow recommendation (*F*(1, 164) = 7.16, *p* < 0.01). A non-significant interaction effect of communication style and service robot type on likelihood to follow recommendation was also detected (*F*(1, 164) = 1.86, *p* = 0.17). Participants who were served by the AN (with anthropomorphic appearance) exhibited a higher likelihood to follow the service robot’s recommendation compared to those who were served by the tablet (without anthropomorphic appearance) (*M*_*tablet*_ = 5.48, *M*_*android*_ = 5.95, *p* < 0 0.01). This finding is encouraging as it validates the significant effect of robot anthropomorphic embodiment on human behaviour, in particular, their likelihood to comply to the robot. We could then confidently confirm our H1.

A two-way ANOVA also revealed a significant main effect of communication style (*F*(1, 164) = 8.02, *p* < 0.05) and a significant main effect of service robot type on perceived mind (*F*(1, 164) = 21.68, *p* < 0.01). A non-significant interaction effect of communication style and service robot type on perceived mind was also detected (*F*(1, 164) = 1.14, *p* = 0.29). Participants who were served by the AN (with anthropomorphic appearance) exhibited a higher perceived mind compared to those who were served by the tablet (without anthropomorphic appearance) (*M*_*tablet*_ = 3.44, *M*_*android*_ = 4.51, *p* < 0 0.01). Participants who were served by a service robot that communicated with voice exhibited a higher perceived mind compared to those who were served by a service robot that communicated with text (*M*_*voice*_ = 4.51, *M*_*text*_ = 3.44, *p* < 0 0.01).

A two-way ANOVA further revealed a non-significant main effect of communication style (*F*(1, 164) = 0.31, *p* = 0.58) and a significant main effect of service robot type on persuasiveness (*F*(1, 164) = 6.37, *p* = 0.01). A non-significant interaction effect of communication style and service robot type on persuasiveness was also detected (*F*(1, 164) = 0.49, *p* = 0.49). Participants who were served by the AN (with anthropomorphic appearance) reported higher persuasiveness compared to those who were served by the tablet (without anthropomorphic appearance) (*M*_*tablet*_ = 4.89, *M*_*android*_ = 5.42, *p* < 0 0.01).

Study 2a overall confirms the positive effect of embodiment of the service robot on willingness of the user to follow recommendations. This study also reveals the positive effect of embodiment of service robot on perceived mind and persuasiveness signalling the potential existence of underlying mechanism between the two main variables which will be investigated in Study 2b. The finding that shows a service robot that communicated with voice is perceived to possess higher perceived mind compared to the one that communicated with text indicates that voice is an important attribute, which itself can provide embodied service robots with human-like qualities. This finding is aligned with^19^.

### Study 2b

#### Method and procedure

We recruited 122 participants over 18 years of age through flyers distributed within Monash University, Australia and invitations on social media (*M*_*age*_ = 27.8 years, *SD* = 9.28, 54.9% male). Based on the results of Study 1, PT was omitted from the study and PV was used as the low anthropomorphised robot and AN as the high anthropomorphised robot in Study 2b, resulting in 2 (robot type: PV vs. AN) × 2 (language style: emotive vs. informative) between-subjects experimental design. The experiment procedure is the same as Study 1.

#### Measures

Similar to previous studies, to validate our service robots’ anthropomorphic manipulation, participants indicated whether the robot they interacted with felt like a person (*M* = 4.02, *SD* = 1.74). Participants were then asked to evaluate the warmth of the robot as another indicator of anthropomorphism (*M* = 4.94, *SD* = 1.19, *α* = 0.87). To validate our service robots’ language style manipulation, participants were asked to indicate the extent to which the service robot communicated with more informational or emotional language style (1 = strongly informational to 7 = strongly emotional) using one item adapted from^57^ (*M* = 3.33, *SD* = 1.87). Participants were asked to indicate their agreement to one item measuring their likelihood to follow recommendation by the service robot (*M* = 5.49, *SD* = 1.36). In addition, they were asked to indicate their perceived mind towards the service robot using four items adapted from^33^ (1 = Not at all to 7 = Very much*; M* = 3.81, *SD* = 1.43, *α* = 0.83) and their evaluation towards the service robot’s persuasiveness using one item (*M* = 5.05, *SD* = 1.28). All the other items were measured on a seven-point scale (1 = Strongly Disagree to 7 = Strongly Agree). Finally, participants were asked to rate whether they believed that the scenario presented to them was realistic (1 = Not at all realistic, 7 = Very realistic; M = 5.43, SD = 1.39), with no difference between conditions (*F*(1, 118) = 0.22, *p* = 0.64). Appendix B lists all items used in this study. For constructs that were measured by multiple items, we created an overall index by taking the average of all items measuring that particular construct and used this overall index in our data analysis.

#### Analysis and results

##### Manipulation check

A one-way ANOVA revealed a significant difference between the two *language style* conditions, with participants in the informational language style condition reporting a lower score in perceived language style than those in the emotional language style condition (*M*_*informational*_ = 2.46, *M*_*emotional*_ = 4.15, *F*(1, 119) = 32.27, *p* < 0.001); confirming the successful manipulation of language style of the service robot.

A one-way ANOVA revealed that participants reported significantly different levels of perceived anthropomorphism (robot felt like a person) for the two-service robot behaviour stimuli (F (1,119) = 46.76, p < 0.001). Pairwise comparisons indicated that the mean score for AN (*M* = 4.92, *SD* = 1.46) was significantly different from PV (*M* = 3.08, *SD* = 1.49). A one-way ANOVA further revealed that participants reported significantly different levels of perceived warmth as an indicator of anthropomorphism for the two service robot behaviour stimuli. Participants interacting with the Pepper robot reported a lower score in perceived anthropomorphism than those interacting with the android robot (*M*_*PV*_ = 4.41, *M*_*AN*_ = 5.44, *F*(1, 119) = 27.66, *p* < 0.001); confirming the successful manipulation of behaviour of the service robot.

##### Hypothesis testing

A two-way ANOVA revealed a non-significant main effect of language style (F(1, 118) = 1.78, p = 0.19) and a significant main effect of service robot type on perceived mind (F(1, 118) = 10.24, p < 0.01). A non-significant interaction effect of language style and service robot type on perceived mind was also detected (F(1, 118) = 0.005, p = 0.94). Participants who were served by the AN exhibited a higher perceived mind compared to those who were served by the PV (M_*PV*_ = 3.40, M_*AN*_ = 4.20, p < 0 0.01).

A two-way ANOVA revealed a non-significant main effect of language style (F(1, 118) = 1.01, p = 0.32) and a significant main effect of service robot type on persuasiveness (F(1, 118) = 7.57, p < 0.01). A non-significant interaction effect of language style and service robot type on perceived mind was also detected (F(1, 118) = 0.50, p = 0.48). Participants who were served by the AN reported higher persuasiveness compared to those who were served by the PV (M_*PV*_ = 4.73, M_*AN*_ = 5.36, p < 0.01).

A two-way ANOVA revealed a non-significant main effect of language style (F(1, 118) = 0.18, p = 0.68) and a non-significant main effect of service robot type on likelihood to follow recommendation (F(1, 118) = 0.21, p = 0.65). While the non-significant main effect of language style on likelihood to follow recommendation is consistent with Study 1, the non-significant main effect of service robot type on likelihood to follow recommendation is inconsistent with Study 1. Nevertheless, the direction of this main effect is as predicted. That is, participants who were served by the AN reported higher likelihood to follow recommendation compared to those who were served by the PV (M_*PV*_ = 5.53, M_*AN*_ = 5.64, p = 0.65). While not as expected, this finding can also be interpreted as the mediating factors are needed to better understand the underlying mechanism between service robot type and likelihood to follow recommendation, which is the aim of Study 2. To complement this finding, we ran a simple regression using SPSS Statistics 27.0, with perceived warmth (a surrogate measure of anthropomorphism) as the independent variable and likelihood to follow recommendation as the dependent variable. Estimation of the simple regression revealed a significant positive effect of perceived warmth on likelihood to follow recommendation (*b* = 0.32, SE = 0.09, p < 0.001), indicating that when users perceive the service robot as warmer (i.e. a higher anthropomorphised service robot), the more likely they will follow its recommendation. This finding corroborates our prediction of the relationship between these two variables.

A non-significant interaction effect of language style and service robot type on likelihood to follow recommendation was also detected (F(1, 118) = 1.20, p = 0.28). This further confirmed that language style does not influence the relationship between service robot type and likelihood to follow recommendation. Thus, in our further serial mediation analysis, language style is used as our control variable.

**Figure 3:**
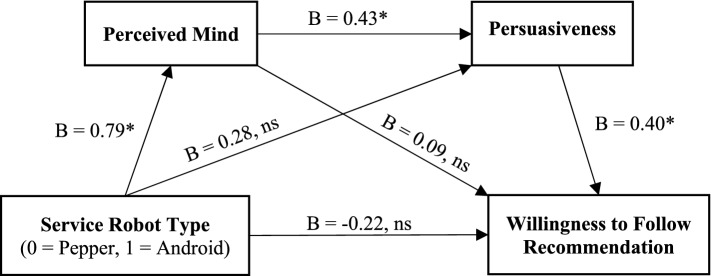
Study 2b: sequential mediation of service robot type on willingness to follow recommendation through perceived mind and persuasiveness (controlled for language type). *p < 0.01.

To test H2, we utilized^61^’s PROCESS macro (Model 6) with 10,000 bootstrap samples. In running this serial mediation analysis, we included service robot type as our independent variable and likelihood to follow recommendation as our dependent variable with the two mediators: perceived mind and persuasiveness (while controlling for the language type). The results revealed a significant serial mediation effect (B = 0.14, 95% CI 0.04, 0.28). As Fig. 3 illustrates, participants interacting with the android robot evaluate such robot to possess greater perceived mind (B = 0.79, *p* < 0.01) than those interacting with the Pepper robot, leading to higher perceived persuasiveness (B = 0.43, *p* < 0.01) and consequently greater likelihood to follow recommendation by the android robot (B = 0.40, *p* < 0.01). Including the perceived mind as a single mediator in the model (Service robot type → Perceived Mind → Willingness to follow recommendation) did not yield any significant indirect effect (B = 0.08, 95% CI − 0.07, 0.25). This is also the same case when including persuasiveness as a single mediator in the model (Service robot type → Perceived Mind → Willingness to follow recommendation; B = 0.11, 95% CI = –0.05, 0.31). Moreover, reversing the mediators such that persuasiveness preceded perceived mind (Service robot type → Persuasiveness → Perceived Mind → Willingness to follow recommendation) failed to uncover any significant indirect effects (B = 0.03, 95% CI − 0.03, 0.12). H2 is thus supported.

## Discussion

### Findings and implications

To the best of our knowledge, this study is the first to investigate the effect of the level of anthropomorphism and language style, as well as the sequential mediation effect of perceived mind and persuasiveness on the customer’s willingness to follow recommendations.

Findings indicate that participants could not differentiate the PV and PT robot types, implying that PV and PT were perceived similarly despite the addition of speech and gestures in PV. This may be due to the fact that head tilts and gestures occur with little simultaneous eye contact^40^ and the perceived authenticity of non-verbal cues is limited^62^. This also supports that the addition of speech does not necessarily affect customer impressions and behaviours^63^. Hence, participants focused merely on the appearance of the PV and PT robot types rather than the behaviours of them.

In addition, although participants were able to deduce emotive language from informative language, there was no effect of the language type on the participant’s willingness to follow recommendations. This result contradicts prior research which showed the significant influence of language type on customer evaluations in a service setting^25^. It might be due to the different definition of language style in this study compared to previous research, as well as the different experiment setup we have used in this study. In addition, participants nowadays are used to devices that use human-like language (e.g. Siri, Google) and may not perceive the voice in PV as a significant feature compared to the tablet in PT.

Findings indicating that participants perceive tablet screen (without anthropomorphic appearance) differently from the AN (with anthropomorphic appearance) further confirm the positive influence of an anthropomorphic representation of the service robot on users’ willingness to follow the robot’s recommendation. Communication style of service robot (text vs. voice) was found to be not an influencing factor on users’ willingness to follow the robot’s recommendation. While this is beyond the scope of our study, our reasonings behind this finding are either participants have no expectations of service robot’s communication style (i.e. they reasonably accept service robots that communicate through either text or voice as both of these communication styles are still relatively commonly observed in real life) or the experiment set up was not conducive enough to trigger any differing effects on this variable. Nevertheless, it is important to note that we found that service robots that communicated with voice are perceived to be higher in perceived mind compared to those communicating with text only. Voice could then be an important human-like attribute that can influence users’ perception of service robot anthropomorphism and later their subsequent behaviours.

Overall Study 1 and Study 2b confirm both H1 and H2. The positive effect of the service robot’s anthropomorphic features on the participant’s willingness to follow recommendations is in line with previous findings about the positive effect of social agency on compliance^64^, and increased willingness to reengage with robots with higher anthropomorphism^45^.

Results show that two mediating variables in a specific sequence are required to significantly influence participant’s willingness to follow recommendations. Therefore, an indirect effect of anthropomorphism level on the willingness to follow recommendations through perceived mind followed by persuasiveness is present. Compared to the PV, the AN with a higher anthropomorphism level is seen as having a higher perceived mind. This is due to AN communicating through a human voice and incorporating several gestures and facial expressions. As a result, participants may perceive the robot as being intelligent and able to deduce their thoughts and feelings to some extent^40,65^. Therefore, the persuasiveness of the AN increases which in turn intensifies the participant’s willingness to follow its recommendations.

This study contributes to the improvement of any human–robot interaction aimed at persuading the customer to follow the robot’s recommendations. Findings show that while language style does not affect the outcome, the human-like attributes of the agent including its appearance, voice and gestures indirectly affect the decision of the customer through their perception of the agent and its persuasiveness. The mediating effect of perceived mind and persuasiveness can be further explored by manipulating other variables that affect these two factors such as speaking in one’s native language.

### Limitations and future work

Our study focused only on a service setting in the tourism industry. We encourage future research to replicate the results in other settings where following the service provider’s recommendations by the customer is used as a measure of success of the service. Potential HRI scenarios may be in the healthcare and education, where the patient or student are advised to follow the clinician or teacher’s advice for an improved health or learning. Unlike the tourism sector, frequent long-term interaction with the support staff in healthcare and education sector is quite common. These scenarios will also pave the path for longitudinal studies and further insight on the influence of novelty or habituation effect^66^ on following recommendations.

This study shows that different levels of service robot anthropomorphism influence the willingness of users to follow their recommendations through perceived mind and persuasiveness. It is pertinent to be mindful that we manipulated the different levels of service robot anthropomorphism using a combination of attributes: physical embodiment, communication style, movement, and facial gestures. This approach fits with the objective of our study as our focus is on investigating service robots that exhibit different levels of anthropomorphism holistically. Future research may replicate this approach by introducing different combinations of attributes to further validate our tested relationships. Alternatively, future research may investigate the effect of each attribute utilized in this study on our tested relationships.

In addition, one intriguing finding observed from this study is that a service robot that communicated with voice is perceived to have higher perceived mind compared to a service robot that communicated with text, while communication style (voice vs. text) is found to have no effect on willingness to follow recommendations. This signals the potential of the perceived mind as the mediating variable between service robot’s communication style and willingness to follow recommendations. Future research could further explore this relationship.

Furthermore, to measure perceived mind, we selected four descriptors from the work of^33^ that we considered best in representing our study context. Hence, our findings need to be carefully interpreted and may not be generalised to other contexts in which other descriptors are more relevant. Moreover, due to the COVID-19 related pandemic restrictions, this study was conducted online. Authors acknowledge the inherent limitations of this approach compared to in-person interaction with real robots that interact by performing gestures and react to the user's movements. This is especially true in case of the high levels of anthropomorphism described for the AN. Investigating the effect of robot type on willingness to follow recommendations through in-person studies will provide further insight. The fact that communication style and language style did not have any effect may be also due to lack of personal interaction with the actual robots. Hence, findings related to communication style and language style need to be carefully interpreted as in-person studies may be required to further confirm them.

Finally, although most of the participants have had previous experience with the Zoom online platform, occasional lack of familiarity hindered the smooth implementation of the study in a number of cases. Additionally, as the human–robot interaction strictly follows a scripted scenario, there might be a decrease in persuasiveness and perceived mind as participants’ interests are not considered. Also, participants in our study are mostly from the younger cohort with engineering background. Recruiting from a more diverse pool of subjects will provide a deeper understanding of customers’ behaviour in interaction with service robots.

## Conclusion

This experimental study is the first to investigate the effect of service robot’s type and its language style on customer’s willingness to follow its recommendations. The findings provide valuable information on the factors that affect the human–robot interaction in service settings. Our findings emphasis the effect of anthropomorphism but remove the focus from the language style. We highlighted the significance of perceived mind and persuasiveness and detailed their effect through a novel sequential mediation model. This provides a better understanding of HRI in service settings and ways to improve customer’s experience.

Findings of this study are particularly useful in service where following the agent’s recommendations is used to assess the success of the HRI, such as many scenarios in the tourism industry, healthcare, and education.

## Supplementary Information

Supplementary Information.

## Data Availability

The datasets generated during and/or analysed during the current study are available from the corresponding author on reasonable request.
