# Supplementary material for: A study on the influence of service robots’ level of anthropomorphism on the willingness of users to follow their recommendations
Source: Sci Rep. 2022 Sep 10;12:15266. doi: 10.1038/s41598-022-19501-0 (PMC9463504; doi:10.1038/s41598-022-19501-0)
Supplement: Supplementary file 1 — Supplementary Information. [file 41598_2022_19501_MOESM1_ESM.pdf]

A study on the influence of service robots' level of anthropomorphism on the willingness of users to follow their recommendations

Elahe Abdi[1*], Dewi Tojib[2], Alexander Kenwa Seong[1], Yamika Pamarthi[1], George Millington-Palmer[1]

[1]*Department of Mechanical and Aerospace Engineering, Monash University, Melbourne, 3800, Australia*

[2]*Department of Marketing, Monash University, Melbourne, 3800, Australia*

**elahe.abdi@monash.edu*

Appendix A – Stimuli for Study 1 and Study 2

Imagine you were in the following situation:

You just arrived in a city you have never been before. You are in your hotel room and now wondering what to explore in this city tomorrow. You only have one day to explore this city. To make the most of your short stay in this city, you decide to ask the hotel concierge. The concierge provides recommendation about three touristic places.

The interaction with the hotel concierge:

Alex greets the participant with a smile and politely says:

"Hello, I am Alex."

"Welcome to our hotel. What can I do for you today?"

☑*I would like to get some ideas on places to explore in this city.**
☐*I would like to find the opening hours of the closest mall around here.*
☐*I would like to find out whether the gym and pool facilities in this hotel are open 24 hours.*

Then Alex says excitedly:

"Okay, can you tell me how many days you will be staying in this city?"

☑*One day*
☐*Two days*
☐*Three days*

Then Alex says passionately:

<table>
<tr><td colspan="2">“Excellent!”
“This city has a lot to explore. Since you only have one day, let me present the top three places you must visit in this city.”
Alex continues saying:</td></tr>
<tr><td>Informative language style</td><td>Emotive language style</td></tr>
<tr><td>“The three things you must visit while you are here:”
“First, the Enchanted Bridge. It is a historical bridge surrounded by forests and mountains.”
“It is the only attraction here that has been voted as a five stars tourist spot by the national tourism body. More than 90% of tourists coming to this town visit the bridge.”
“Second, the Charm Market. This is the best place to discover a wide variety of fresh, quality products and shopping for local arts.”
“Last year alone, this market was posted in social media more than 1 million times and liked by almost 2 million people worldwide.”
“Third, finish your day trip in our best roof top bar ‘Good Heavens’ in the downtown area.”
“It is a place to energise and chill out with delicious local food. This place has won the best local food award by the national culinary club for 10 years in a row.”</td><td>“The three things you must visit while you are here:”
“First, the Enchanted Bridge. It is a historical bridge surrounded by forests and mountains.”
“Walking across the bridge is such a delight. You will enjoy the sun and the blue sky. The soothing sound of waterfall with the pure sound of birds singing will definitely relax your mind, body, and soul.”
“Second, the Charm Market. This is the best place to discover a wide variety of fresh, quality products and shopping for local arts.”
“A very pleasant place to wander. The smell of fresh bread from the oldest bakery will forever stay with you. The way the stalls – fresh food, fruits, flowers, and local arts – display their products will be a feast for your eyes. Once you are there, you will be lost in time.”
“Third, finish your day trip in our best roof top bar ‘Good Heavens’ in the downtown area.”
“It is a place to energise and chill out with delicious local food. You will be</td></tr>
</table>

	**spoiled for choice of mouth-watering local food here. Friendly staff, excellent service, and extraordinary food – these all will guarantee a highly memorable visit.”**
Alex continues saying:<br>“I hope you find this itinerary useful. Would you like me to email this itinerary to you?”<br>☐*Yes*<br>☐*No*<br>“Thank you for dropping by. I hope you have a pleasant stay in our hotel and in this city.”<br>* Participant’s response options are present in *italic font*. The interaction continues only if the participant chooses the right answer based on the explanatory statement.	

Appendix B – Items Used in Study 1 and Study 2

**Constructs and their respective items**
**Anthropomorphism**<br>• The concierge feels like a person [Strongly Disagree: Strongly Agree]
**Anthropomorphism**<br>• The concierge is friendly. [Not at all : Very much]<br>• The concierge is sociable. [Not at all : Very much]<br>• The concierge is likeable. [Not at all : Very much]<br>• The concierge is warm. [Not at all : Very much]<br>• The concierge is kind. [Not at all : Very much]
**Language style**

• The way Alex (the concierge) recommended the top three places is [Strongly informational : Strongly emotional]
**Perceived mind***<br><br>• To what extent do you think Alex is able to host engaging conversations? [Not at all : Very much]<br>• To what extent do you think Alex is able to convey thoughts or feelings to others? [Not at all : Very much]<br>• To what extent do you think Alex is able to have experiences and being aware of things? [Not at all : Very much]<br>• To what extent do you think Alex is able to understand how others are feeling? [Not at all : Very much]
**Persuasiveness***<br><br>• How would you evaluate Alex (the concierge)? [Not at all persuasive : Very much persuasive]
**Follow recommendations**<br><br>• Please indicate how likely you would be to follow the one-day itinerary provided by Alex (the concierge). [Not at all : Very much]
**Realistic scenario**<br><br>• In my opinion, the scenario I participated earlier was [Very unrealistic : Very realistic]

*) Only measured in Study 2a and Study 2b
